# The association between bone health indicated by calcaneal quantitative ultrasound and metabolic syndrome in Malaysian men

**DOI:** 10.1186/s40200-015-0136-3

**Published:** 2015-03-07

**Authors:** Kok-Yong Chin, Soelaiman Ima-Nirwana, Isa Naina Mohamed, Fairus Ahmad, Elvy Suhana Mohd Ramli, Amilia Aminuddin, Wan Zurinah Wan Ngah

**Affiliations:** Pharmacology Department, Universiti Kebangsaan Malaysia Medical Centre, Jalan Yaacob Latif, Bandar Tun Razak 56000 Cheras, Kuala Lumpur, Malaysia; Anatomy Department, Universiti Kebangsaan Malaysia, Medical Centre, Kuala Lumpur, Malaysia; Physiology Department, Universiti Kebangsaan Malaysia, Medical Centre, Kuala Lumpur, Malaysia; Biochemistry Department, Faculty of Medicine, Universiti Kebangsaan Malaysia, Kuala Lumpur, Malaysia

**Keywords:** Bone, Calcaneal, Men, Metabolic syndrome, Quantitative ultrasound

## Abstract

**Background:**

Previous studies on the relationship between bone health and metabolic syndrome (MS) have revealed heterogeneous results. There are limited studies employing bone quantitative ultrasonometry in evaluating this relationship. This study aimed to determine the relationship between MS and bone health in a group of Malaysian middle-aged and elderly men using bone quantitative ultrasonometry.

**Methods:**

This cross-sectional study recruited 309 free living Chinese and Malay men aged 40 years and above residing in Klang Valley, Malaysia. Their demographic and anthropometric data were collected. Their calcaneal speed of sound (SOS) was measured using a CM-200 bone ultrasonometer. Their blood was collected for the evaluation of lipid profile, total testosterone and sex hormone-binding globulin. The joint interim MS definition was used for the classification of subjects. Multiple linear regression analysis was used to assess the association between SOS and indicators of MS and the presence of MS, with suitable adjustment for confounders.

**Results:**

There was no significant difference in SOS value between MS and non-MS subjects (p > 0.05). The SOS values among subjects with different MS scores did not differ significantly (p > 0.05). There were no significant associations between SOS values and indicators of MS or the presence of MS (p > 0.05).

**Conclusions:**

The relationship between bone health and MS is not significant in Malaysian middle-aged and elderly men. A longitudinal study should be conducted to evaluate the association between bone loss and MS to confirm this finding.

## Introduction

Osteoporosis and cardiovascular diseases constitute two major health problems worldwide [1]. Metabolic syndrome (MS) is a cluster of metabolic phenotypes, which in a whole predicts cardiovascular events better than the sum of each indicator [2]. Several MS definitions have been put forward by various organizations, such as the World Health Organization [3], the International Diabetes Federation [4], and the National Cholesterol Education Program Adult Treatment Panel III criteria [5]. Recently, a joint interim statement has been introduced to harmonize the MS criteria [6]. Briefly, these criteria include central obesity, increased blood pressure (BP), triglyceride (TG) and fasting blood glucose (FBG) levels and decreased high-density lipoprotein cholesterol (HDL) level [6].

Deterioration of bone health is a direct consequence of aging in both men and women [7], but less attention is given to men [8]. Previous studies have found that each MS indicators is related to bone health in humans. Obesity increases mechanical loading and is related to improved bone health [9]. Proinflammatory cytokines, leptin and adiponectin secreted by adipocytes can influence bone metabolism [10]. Hyperlipidemia has been shown to increase resorption activity in the bone [11] and lipid peroxidation products can induce oxidative damage on bone-forming osteoblasts [12]. Hypertension increases parathyroid hormone level and urinary calcium excretion [13,14]. Type I diabetes increases bone mineral density while type II diabetes decreases it [15].

The presence of metabolic syndrome has been associated with variations in bone mass measured by dual-energy X-ray absorptiometry (DEXA) and calcaneal quantitative ultrasonometry (QUS) in men. However, the results obtained are heterogeneous, whereby positive [16,17], negative [18] and non-significant [19,20] associations have been reported. Due to the limited number of studies available, no conclusive statement can be drawn on whether MS exerts beneficial or adverse effects on bone health in men.

This study aimed to examine the relationship of MS on bone health in a group of Malaysian Chinese and Malay men. The calcaneal QUS technique was used because literatures on the relationship between MS and bone QUS indices in men were limited. We hope that this study would provide more evidence on the clinical impact of MS on bone health in men, which is an issue deserving more attention.

## Material and methods

The current study was conducted as part of the Malaysian Aging Male Study [21,22]. The recruitment was performed between September 2009 and September 2011. The subjects recruited were Malaysian Chinese and Malay males, aged 40 years and above, residing in Klang Valley, Malaysia. The subjects were invited via advertisements in major newspapers, radio broadcasts, flyers, public announcements in community centers and religious places. The inclusion and exclusion criteria were mentioned clearly in the invitation. The following subjects were excluded from this study: (1) those suffering from chronic bone diseases such as osteoporosis, osteomalacia, Paget’s disease etc.; (2) those undergoing treatment or taking medication that might alter bone metabolism, such as sex hormone replacement, thyroid supplements, thiazide diuretics, lithium, glucocorticoids etc.; (3) those with mobility problems and need walking aids; (4) those undergoing a major surgery or suffering from a fracture six months prior to the screening; (5) those unable to complete the screening process. The subjects participated on their own volition. They were briefed on the details of this study and written consent was obtained before the enrollment. The Research and Ethics Committee of Universiti Kebangsaan Malaysia Medical Center reviewed and approved the study protocol (Code: UKM-AP-TKP-09-2009).

The subject answered a detailed questionnaire on their demographic characteristics. Their age was determined from the records on their identification cards and their ethnicity was self-declared. They underwent a physical examination performed by qualified physicians and their medical history was taken.

The height of the subjects without shoes was determined using a portable stadiometer (SECA, Hamburg, Germany) and was recorded to the nearest 0.1 cm. The weight of the subjects with light clothing without shoes were determined using a weighing scale (Tanita, Tokyo, Japan) and was recorded to the nearest 0.1 kg. Their body mass index (BMI) was calculated as per the convention. Their waist circumference (WC) was measured using a soft measuring tape midway between the lower rib margin and the superior border of the iliac crest at the end of a normal expiration in the standing position. It was recorded to the nearest 1 cm. Their calcaneal speed of sound (SOS) was measured using the CM-200 bone ultrasonometer (Furuno, Nishinomiya, Japan). Calibration of the device was carried out at the beginning of each screening session and the measurement was performed by a trained technician. Three readings were taken per subject and the average SOS value was used in the analysis. The short-term *in vivo* precision of the bone ultrasonometer was 0.19%. The blood pressure (BP) of the subjects was determined using a mercury sphygmomanometer in the sitting position. If the blood pressure was high, the subjects were requested to rest for 15 minutes before another reading was taken.

All subjects were required to fast for at least eight hours before blood collection. Venipuncture was performed by qualified phlebotomists or physicians between 0830 and 1030 hours. Serum was extracted immediately after the blood collection. Part of the serum was sent immediately to an accredited laboratory for lipid profile and total testosterone (TT) evaluation while the rest was stored at -80°C until analysis. The ADVIA 2400 Chemistry Analyzer (Siemens Healthcare Diagnostics, Illinois, USA) was used to evaluate total cholesterol (TC), triglyceride (TG) and high-density lipoprotein cholesterol (HDL) levels using enzymatic method. The ADVIA Centaur immunoassay system (Siemens Healthcare Diagnostics, Illinois, USA) was used to evaluate TT level using direct chemiluminescent technique. Enzyme-linked immunobsorbent kit (IBL International, Hamburg, Germany) was used to evaluate the level of sex hormone-binding globulin (SHBG). The ACCUCHEK portable glucometer (Roche, Basel, Switzerland) was used to measure fasting blood glucose (FBG) level by the glucose oxidase method.

### Definition of MS

Metabolic syndrome was defined using the harmonized criteria based on the joint interim consensus [6]. Subjects fulfilling at least three out of the five criteria listed were categorized as MS subjects: (1) WC ≥ 90 cm; (2) TG ≥ 1.7 mmol/l or on drug treatment for elevated TG; (3) HDL ≤ 1.0 mmol/l or on drug treatment for reduced HDL; (4) Systolic BP ≥ 130 mmHg and/or diastolic BP ≥ 85 mmHg or on drug treatment for hypertension; (5) FBG ≥ 5.6 mmol/l or on drug treatment for elevated blood glucose.

### Statistical analysis

The normality of the data was assessed using Shapiro-Wilk test with the aid of histograms. Skewed data were log-transformed and used in the analysis. The level of FBG could not be normalized; hence it was analyzed using non-parametric tests. The comparison of the basic characteristics of the subjects was performed using independent t-test for normally distributed data and Mann-Whitney U test for FBG. The comparison of SOS values among the groups based on their MS scores (0-5) was made using a general linear model (univariate) with adjustment for confounders. The associations between SOS and MS, and each indicator of MS were evaluated using multiple linear regression analysis. The indicators of MS were first entered as continuous variables and then as dichotomous variables (1 = the presence of a condition/0 = the absence of a condition) into the regression models. The fasting blood glucose level was entered into the regression models in dichotomous form because the data were skewed. In a separate regression analysis, the presence or absence of MS was entered as a dichotomous variable. For all the regression analyses performed, three models were generated: model 1 contained only the MS indicators or the presence of MS; model 2 contained the predictors in model 1 and was adjusted for confounders such as age, ethnicity, smoking status, TT and SHBG levels; model 3 contained the predictors in model 2 and was further adjusted for BMI. The significant value for all analysis performed was set at p<0.05. Statistical Package for Social Sciences version 16 (SPSS Inc., Chicago, USA) was used to perform all the analysis.

## Results

A total of 333 subjects with complete metabolic phenotypes, calcaneal SOS, serum TT and SHBG data were eligible for this study. After eliminating statistical outliers, data from 309 subjects (age range 39-81 years; mean 54.05 years) were used in the analysis.

The prevalence of MS was 61.5% (190 subjects) in the study population. Subjects with MS were significantly older, heavier and had higher BMI compared to subjects without MS (p<0.05). The WC, systolic BP, diastolic BP, TG, FBS and SHBG levels of the MS subjects were significantly higher compared to the non-MS subjects (p<0.05). The HDL and TT levels of the MS subjects were significantly lower compared to the non-MS subjects (p<0.05). The two groups did not show significant differences in height, calcaneal SOS, TC and LDL levels (p>0.05). Smoking status and ethnicity were not related to MS (p>0.05) (Table 1).

**Table 1:** **The comparison of basic characteristics between MS and non-MS subjects**

	**MS (n=190)**		**Non-MS (n=119)**		
	**Mean**	**SD**	**Mean**	**SD**	**p-value**
Age (years)	55.46	8.86	51.80	9.72	**0.001**
Height (m)	1.67	0.06	1.67	0.06	0.843
Weight (kg)	72.70	10.19	65.61	11.01	**<0.001**
BMI (kg/m^2^)	26.03	3.61	23.77	3.75	**<0.001**
SOS (m/s)	1511.70	24.12	1513.80	24.04	0.451
WC (cm)	99.70	6.05	95.70	7.80	**<0.001**
Bpsys (mmHg)	146.88	16.72	132.15	16.46	**<0.001**
Bpdia (mmHg)	87.48	9.97	80.79	9.53	**<0.001**
HDL (mmol/l)	1.22	0.25	1.40	0.29	**<0.001**
TG (mmol/l)	1.85	0.82	1.13	0.50	**<0.001**
TC (mmol/l)	5.70	1.00	5.72	0.85	0.705
LDL (mmol/l)	3.64	0.90	3.79	0.81	0.066
FBS (mmol/l)	6.43	1.76	5.46	0.63	<0.001*
TT (nmol/l)	17.56	5.95	21.00	7.55	**<0.001**
SHBG (nmol/l)	47.31	24.72	56.10	31.75	**0.008**
Smoker (n)	32	-	27	-	0.203^†^
Non-smoker (n)	158	-	92	-	
Malay (n)	53	-	30	-	0.604^†^
Chinese (n)	137	-	89	-	

*Indicates that Mann-Whitney U test is used for analysis. ^†^indicates that Pearson’s Chi-square is used for analysis. For all other data, independent t-test is used for analysis.

*Abbreviation:* *BMI* Body mass index, *Bpdia* diastolic blood pressure, *Bpsys* Systolic blood pressure, *FBG* fasting blood glucose, *HDL* high density lipoprotein cholesterol, *LDL* low-density lipoprotein cholesterol, *SD* standard deviation, *SHBG* sex hormone-binding globulin, *SOS* calcaneal speed of sound, *TC* total cholesterol, *TG* triglycerides, *TT* total testosterone, *WC* waist circumference.

There were no significant differences in SOS values among subjects with different MS scores (0 to 5) (p>0.05). Further adjustment for age, BMI, ethnicity, TT and SHBG levels did not change the results (p>0.05) (Table 2).

**Table 2:** **The comparison of SOS values among men with different MS score**

**MS score**	**Adjusted SOS value** ^**†**^ **(m/s)**		**p-value**
	**Mean**	**Std. error**	
0	1519.56	8.58	0.267
1	1514.86	4.19	
2	1511.59	2.72	
3	1509.96	2.19	
4	1517.87	3.11	
5	1505.98	6.92	

^†^The SOS values are adjusted for age, BMI, ethnicity, TT and SHBG levels of the subjects.

*Abbreviation*: *MS* metabolic syndrome, *SOS* calcaneal speed of sound.

Multiple linear regression analysis revealed that indicators of MS were not associated with calcaneal SOS in unadjusted and adjusted models (p>0.05) (Table 3). Reanalyzing the data in dichotomous forms (by considering the presence or absence of the MS indicators) did not change the results (p>0.05) (Table 4). The presence or absence of MS was not associated with calcaneal SOS in the study population (p>0.05) (Table 5).

**Table 3:** **Multiple linear regression results using indicators of MS as continuous variables**

	**Model 1**		**Model 2**		**Model 3**	
**Predictor**	**β**	**p-value**	**β**	**p-value**	**β**	**p-value**
WC	0.069	0.267	0.026	0.702	−0.111	0.236
BPsys^†^	−0.087	0.288	0.041	0.644	0.036	0.679
BPdia	0.113	0.167	0.020	0.815	0.007	0.935
HDL^†^	0.094	0.154	0.107	0.103	0.116	0.076
TG^†^	0.016	0.811	0.067	0.318	0.073	0.273
FBG*	−0.072	0.228	−0.043	0.480	−0.051	0.397

^†^Indicates that log-transformed values are used in the analysis. *indicates that dichotomous values (0=absence/1=presence of elevated FBG) are used in the analysis.

Model 1 includes indicators of metabolic phenotype. Model 2 includes predictors in model 1, age, ethnicity, smoking status, total testosterone and sex hormone-binding globulin levels. Model 3 includes predictors in model 2 and body mass index.

*Abbreviation:* *BMI* body mass index, *BP* blood pressure, *FBG* fasting blood glucose, *HDL* high density lipoprotein cholesterol, *MS* metabolic syndrome, *TG* triglycerides, *WC* waist circumference, *β* standardized coefficient of regression.

**Table 4:** **Multiple linear regression results using indicators of MS as dichotomous variables**

	**Model 1**		**Model 2**		**Model 3**	
**Predictor**	**β**	**p-value**	**β**	**p-value**	**β**	**p-value**
Central adiposity	0.085	0.145	0.030	0.612	−0.008	0.901
Elevated BP	−0.049	0.407	0.002	0.974	−0.056	0.357
Reduced HDL	−0.029	0.612	−0.041	0.479	−0.007	0.911
Elevated TG	0.042	0.481	0.080	0.183	−0.049	0.394
Elevated FBG	−0.072	0.223	−0.043	0.479	0.080	0.183

Model 1 includes the presence (0 = absence/1 = presence) of a metabolic phenotype. Model 2 includes predictors in model 1, age, ethnicity, smoking status, total testosterone and sex hormone-binding globulin levels. Model 3 includes predictors in model 2 and body mass index.

*Abbreviation:* *BMI* body mass index, *BP* blood pressure, *FBG* fasting blood glucose, *HDL* high density lipoprotein cholesterol, *MS* metabolic syndrome, *TG* triglycerides, *WC* waist circumference, *β* standardized coefficient of regression.

**Table 5:** **Multiple linear regression results using the presence of MS as the dichotomous variable**

	**Model 1**		**Model 2**		**Model 3**	
**Predictor**	**β**	**p-value**	**β**	**p-value**	**β**	**p-value**
MS*	−0.043	0.451	0.005	0.934	−0.020	0.743

*Indicates that dichotomous values (0 = absence/1 = presence of metabolic syndrome) are used in the analysis.

Model 1 includes the presence of metabolic syndrome. Model 2 includes predictors in model 1, age, ethnicity, smoking status, total testosterone and sex hormone-binding globulin levels. Model 3 includes predictors in model 2 and body mass index.

*Abbreviation:* *BMI* body mass index, *BP* blood pressure, *FBG* fasting blood glucose, *HDL* high density lipoprotein cholesterol, *MS* metabolic syndrome, *TG* triglycerides, *WC* waist circumference, *β* standardized coefficient of regression.

## Discussion

The current study found that calcaneal SOS did not differ significantly between Malaysian men with and without MS. There was no significant difference in the value of SOS among subjects with different MS scores. None of the MS indicators or MS itself were significantly associated with bone health in Malaysian men as assessed using calcaneal QUS. The results of this study were similar to the findings of Tseng et al. in elderly men and women in Puli Township, Taiwan [20]. They assessed the bone health of the study population using a similar device to ours (Furuno CM-100 ultrasonometer) and discovered that there was no significant difference in SOS values between MS and non-MS subjects [20]. However, they found that an increase in TG and diastolic BP and a decrease in WC were related to an increase in SOS in men, which was not observed in this study [20]. In the Camargo Cohort Study, Hernández et al. found that none of the bone ultrasound indices were significantly different between men with and without MS [19]. They also reported no significant associations between any of the QUS indices and the individual components of MS [19]. The presence of the MS was not significantly related to previous fracture in their study [19]. In a Chinese population, Zhao et al. found that there was no significant difference in estimated BMD (eBMD; a composite bone ultrasound index generated by combining SOS and broadband attenuation of sound; BUA) among male subjects with different MS scores [23]. However, when grouped dichotomously, eBMD was shown to be significantly higher in the MS subjects compared to the non-MS subjects [23]. They also found that an increase in FBG and a decrease in WC and in HDL levels were associated with a significant increase in BMD. These findings were not observed in the current study [23].

The studies on the relationship between MS and BMD in men also showed heterogeneous results. Kim et al. reported that weight-adjusted femoral neck BMD was lower in MS subjects compared to non-MS subjects of both genders [18]. They also found that with increasing MS components, the BMD of the subjects decreased significantly [18]. In American adults, The Third National Health and Nutrition Examination Survey revealed that the femoral neck BMD was significantly higher in MS subjects [17]. The researchers also found that with increasing number in components of MS, the femoral neck BMD of the subjects increased [17]. The positive relationship between MS and BMD was also reported by Boyanov et al. in Bulgarian men [16], Pasco et al. in Australian men [24] and Yamaguchi et al. in Japanese men with type-2 diabetes [25]. Muhlen et al. reported an interesting finding in Rancho Bernardo Study, in which age-adjusted femoral neck BMD was significantly higher in men with MS compared to men without MS [26]. After further adjustment for BMI, the relationship was inverted and MS men were found to have lower BMD [26]. The researchers concluded that the positive association between BMD and MS was attributed to increased BMI in MS subjects [26].

We suggest that the heterogeneity of the results is not due to differences in methodology and site of measurement. Studies using bone QUS at the heel and BMD at the femoral neck had reported positive [17,24], negative [18] and non-significant results [19,20]. Furthermore, incoherent findings were also obtained in populations of similar background (Asian or Western). We suggest that definition of MS, which is originally designated for predicting risk for cardiovascular diseases, is less useful in predicting the risk for reduced bone health status in men. In a meta-analysis, Xue et al. reported that the overall association between MS and femoral neck BMD was not significant, except in the Caucasians [27]. The association between MS and spine BMD was stronger [27]. However, it should be noted that this meta-analysis considered only 11 studies using BMD.

In the current study, we utilized calcaneal SOS as the bone health determinant. Calcaneal QUS was the only bone QUS technique recommended by the International Society of Clinical Densitometry because it was well studied. [28]. The calcaneal bone is a weight-bearing bone of the body [9,29]. Hence, it is sensitive to mechanical loading. The calcaneal bone consists of more than 95% trabecular bone, which provides a high surface per volume ration for the maximal exposure of bone cells to humorous factors [30]. Hence, we suggest that it is a suitable site to detect bone adaptive changes due to mechanical and biochemical variations. Among the numerous MS definitions, the harmonized criteria were used because it is the most updated and suitable for use in the Asian population. We adjusted the analysis in the study for TT, SHBG and BMI because our previous studies showed that these factors were associated with both MS and bone health status in Malaysian men [31-33].

Several limitations should be considered in the interpretation of the results of this study. The study was conducted in Klang Valley and involved Chinese and Malay men only. Hence, the study population might not be representative of the entire Malaysian population. The bone ultrasonometer generated SOS as the bone health determinant, while the other bone ultrasound index, BUA, was not generated. However, a previous study had shown that SOS was more related to BMD and elasticity of the bone [34,35]. Subjects with MS were shown to have higher level of proinflammatory cytokines [36], which could potentially influence bone metabolism. However, proinflammatory cytokine levels were not measured in this study.

## Conclusion

The bone health of middle-aged and elderly men in Malaysia is not significantly associated with MS status and indicators of MS. The usefulness of MS definition in predicting bone health should be reconsidered. A longitudinal study should also be performed to evaluate the relationship between temporal bone loss and MS to confirm the findings.
